# Data supporting the activation of autophagy genes in the diabetic heart

**DOI:** 10.1016/j.dib.2015.09.003

**Published:** 2015-09-14

**Authors:** Pujika Emani Munasinghe, Federica Riu, Parul Dixit, Midori Edamatsu, Pankaj Saxena, Nathan S.J. Hamer, Ivor F. Galvin, Richard W. Bunton, Sharon Lequeux, Greg Jones, Regis R. Lamberts, Costanza Emanueli, Paolo Madeddu, Rajesh Katare

**Affiliations:** a Department of Physiology-HeartOtago, University of Otago, New Zealand; b School of Clinical Sciences, Bristol Heart Institute, University of Bristol, Bristol, United Kingdom; c Department of Cardiovascular Surgery, University of Otago, New Zealand; d Department of Anatomy, University of Otago, New Zealand; e Department of Surgery, University of Otago, New Zealand

## Abstract

This data article contains full list of autophagy related genes that are altered in diabetic heart. This article also shows data from in vitro cultured cardiomyocytes that are exposed the high glucose treatment to simulate hyperglycemic state in vitro. The interpretation of these data and further extensive insights into the regulation of SG biogenesis by AMPK can be found in “Type-2 diabetes increases autophagy in the human heart through promotion of Beclin-1 mediated pathway” (Munasinghe et al., in press) [1].

## Specifications table

**Table:** 

Subject area	Cardiovascular
More specific subject area	Diabetic heart disease
Type of data	*RT-profiler array and in vitro data*
How data was acquired	*RT profiler array uses specific software to do the calculations. in vitro data analysis was done using GraphPad Prism software.*
Data format	*Analyzed*
Experimental factors	*N/A*
Experimental features	*N/A*
Data source location	*Department of Physiology-HeartOtago, Dunedin, New* Zealand and Bristol Heart Institute, Bristol, United Kingdom
Data accessibility	*Data within this article*

## 1 Value of the data

• First RT profiler array data for autophagy genes in the diabetic heart.
• In addition to beclin-1, RT profiler analysis of diabetic heart identified marked changes in several other genes. This could provide a benchmark for future research studies determining the pathophysiological role of other genes in autophagy.
• Isolated adult cardiomyocytes could be a valuable source to study the effect of diabetes in vitro.

## 2 Data, experimental design, materials and methods

### 2.1 Data

**Fig. 1:**
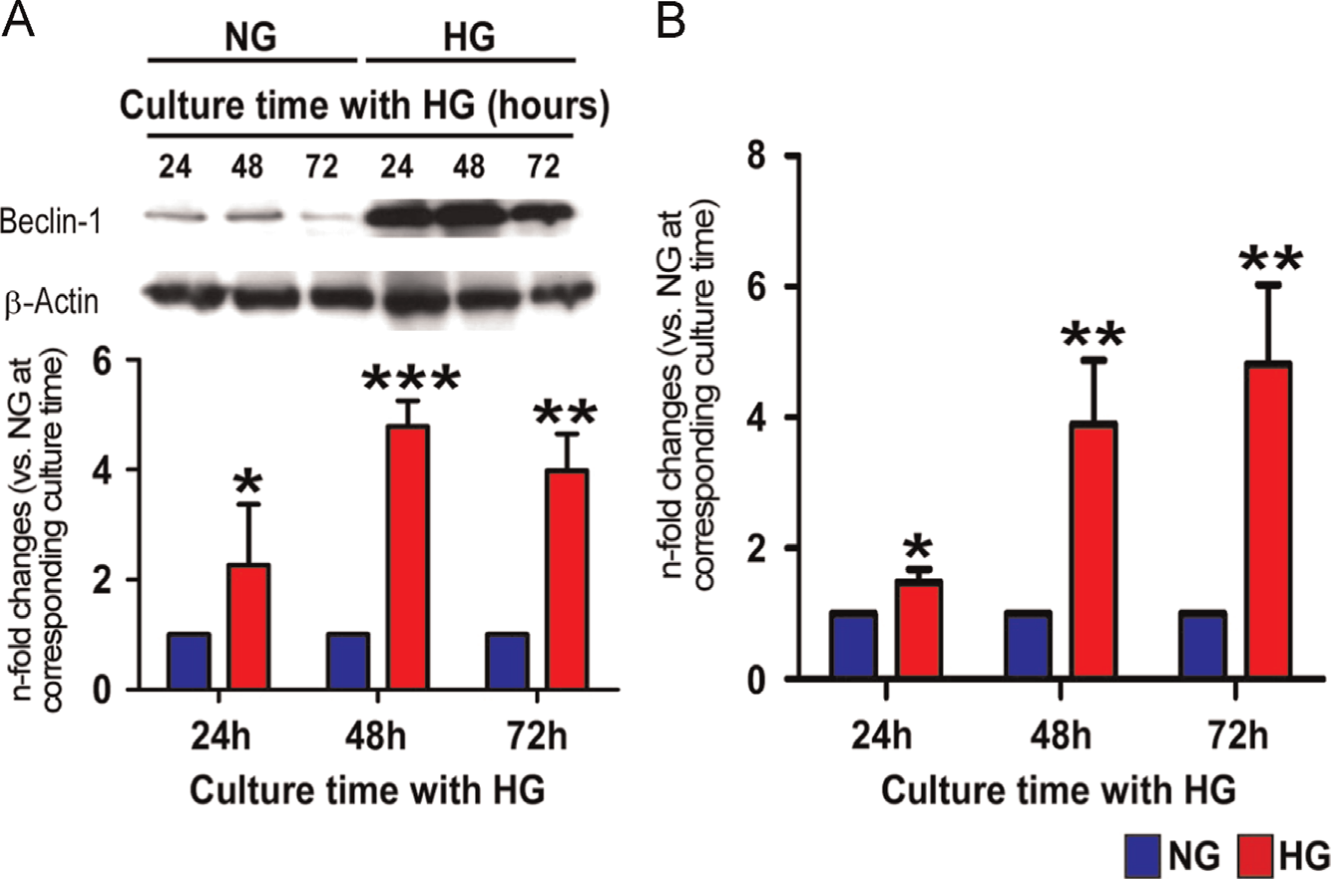
A. Representative blots and bar graphs showing the level of Beclin-1 in rat cardiomyocytes exposed to high glucose (30 mM). B. Bar graphs showing Caspase-3/7 activity in normal and high glucose treated cardiomyocytes at different time points. NG – normal glucose; HG – high glucose. Values are mean±SD of 4 independent experiments and are expressed as fold changes to cells treated with NG at corresponding time point. **P<0.01 and ***P<0.001 vs. NG treated cells at corresponding time point.

RT profiler array showed marked activation of several autophagy related genes with beclin-1 being markedly increased compared to other genes (Table 1). Importantly, exposure of adult cardiomyocytes to high glucose markedly increased the level of beclin-1 within 24 h with a peak increase at 48 h (Fig. 1A). Importantly, the caspase activation which indicates cell death followed the beclin-1 activation (Fig. 1B).

## 3 Experimental design, materials and methods

### 3.1 Animal model of type-2 diabetes

Male obese leptin-receptor mutant BKS.Cg-+Lepr^db^/+Lepr^db^/OlaHsd mice (Harlan, UK) were used as a model of insulin-resistant type-2 DM. Elevations of blood glucose begin at four to six weeks in these mutant mice. Age matched lean mice (BKS.Cg-m+/+Lepr^db^/OlaHsd) were used as control. These mice best represent the human model of type-2 diabetes [2,3].

### 3.2 RNA extraction and RT-profiler array for autophagy genes

Total RNA was extracted from the left ventricle of 8-weeks old diabetic and non-diabetic lean mice using Trizol, according to the manufacturer׳s instructions (Invitrogen, UK). After confirming the purity and integrity of the total RNA, cDNA was prepared from 1 µg of total RNA by Transcription Kit (Qiagen, UK). The activation of apoptotic genes was then evaluated using murine RT-profiler PCR autophagy array (Qiagen, UK) using a light cycler (Roche 480, UK). Data were analyzed using the software package from Qiagen and expressed as fold-changes to control. Fold change of ≥2 was considered significant [1,4,5].

### 3.3 Isolation and culture of adult cardiomyocytes

#### 3.3.1 Isolation and culture of rat adult cardiomyocytes

The male Wistar rats were killed by cervical dislocation, the heart dissected and rinsed in cold solution A containing (in mM): 137 NaCl, 5 KCl, 1.2 MgSO_4_, 1.2 NaH_2_PO_4_, 20 *N*-hydroxyethylpiperazine-*N*′-2-ethanesulphonic acid (HEPES), 16 glucose, 5 Na pyruvate and 1.8 MgCl_2_ (pH 7.25 with NaOH)+0.75 mM CaCl_2_. The heart was cannulated via the aorta and perfused for 4 min with solution A+0.75 mM CaCl_2_ (all perfusing solutions were oxygenated and maintained at 37 °C). This was followed by a 4-min perfusion with solution A+0.09 mM ethylene glycol-*bis* (β-aminoethyl ether) *N*,*N*,*N*,*N*′-tetraacetic acid (EGTA). Next the heart was digested with 50 ml of enzyme solution containing: solution A+0.09 mM EGTA, 50 mg collagenase (Worthington Biochemical Corporation, Lakewood, New Jersey, USA. Type I), 5 mg protease (Sigma, Poole, Dorset, UK. Type IV), with (glutamate loaded) or without (control) 6.4 mM potassium L-glutamate until the tissue felt soft. There was a final 4-min perfusion with solution A+0.15 mM CaCl_2_ before the ventricles were cut down and sliced. The sliced ventricles were suspended in approximately 20–25 ml solution A+0.15 mM CaCl_2_ and shaken for 6 min at 37 °C. After filtration, cells were allowed to sediment, the supernatant was discarded, and the remaining cell layer suspended in solution A+0.5 mM CaCl_2_. This sedimentation, removal of supernatant and resuspension step was repeated, but this time the cells were suspended in solution A+1 mM CaCl_2_. This technique typically produced a yield of over 90% rod-shaped cells with the ability to exclude Trypan Blue [6]. The resulting cells were then washed separately with medium 199 (Invitrogen) supplemented with 0.2% BSA, 10% FBS, 5 mM creatine, 5 mM taurine, 2 mM carnitine, 10 µM cytosine-D-arabinofuranoside (all from Sigma chemicals), ITS and antibiotics (both from Invitrogen). After the final wash cells were resuspended in the same medium and plated on laminin coated culture dish according to the experiments [2].

## 4 Effect of high glucose on beclin-1 expression

After isolation 1×10^6^ cardiomyocytes were seeded on a laminin coated T25 flask and allowed to settle for 4 h. After 4 h cardiomyocytes were exposed to high glucose (HG, 30 mM) or Mannitol (NG, 30 mM for osmotic control) to simulate diabetic condition in vitro for 48 h. After 48 h, the effect of HG treatment on beclin-1 was measured by western blotting [7] and cell survival by caspase-3/7 activity as described earlier [2,8].

## Figures and Tables

**Table 1:** RT profiler assay showing changes in the expression pattern of autophagy associated genes in the type-2 diabetic mouse heart at 12 weeks of age. Activation of the autophagy genes was evaluated using murine RT-profiler PCR autophagy array (Qiagen, UK). RNA from snap frozen mouse hearts (12 weeks of age) was isolated with TRIzol (Invitrogen, UK). One microgram of total RNA was reverse transcribed and resulting cDNA was amplified in a light cycler (Roche 480, UK). Data were analyzed using the software package from Qiagen and expressed as fold-changes to non-diabetic. Genes showing a fold change of ≥2 and a *T*-test of <0.05 were considered to be significantly modulated.

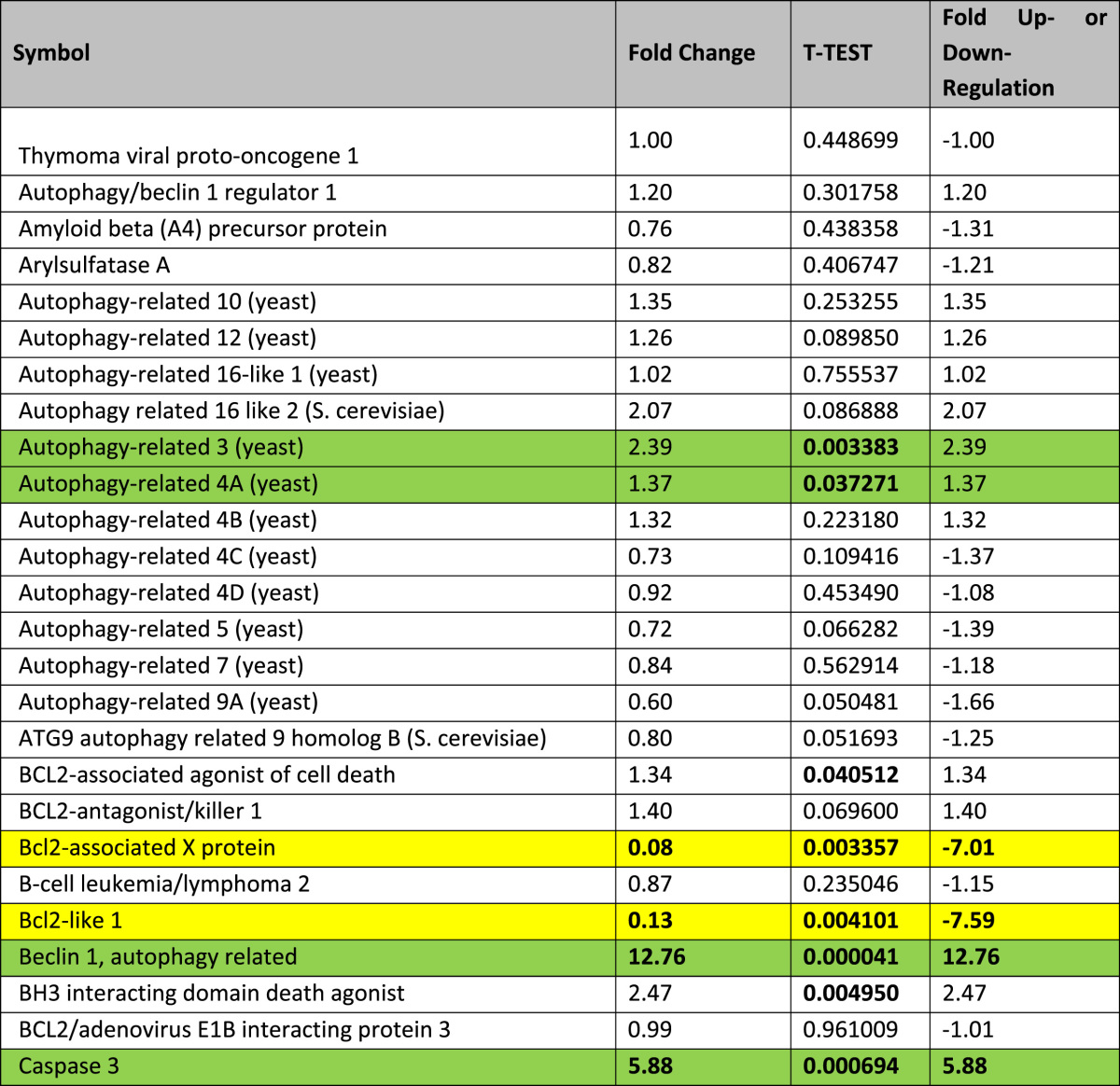
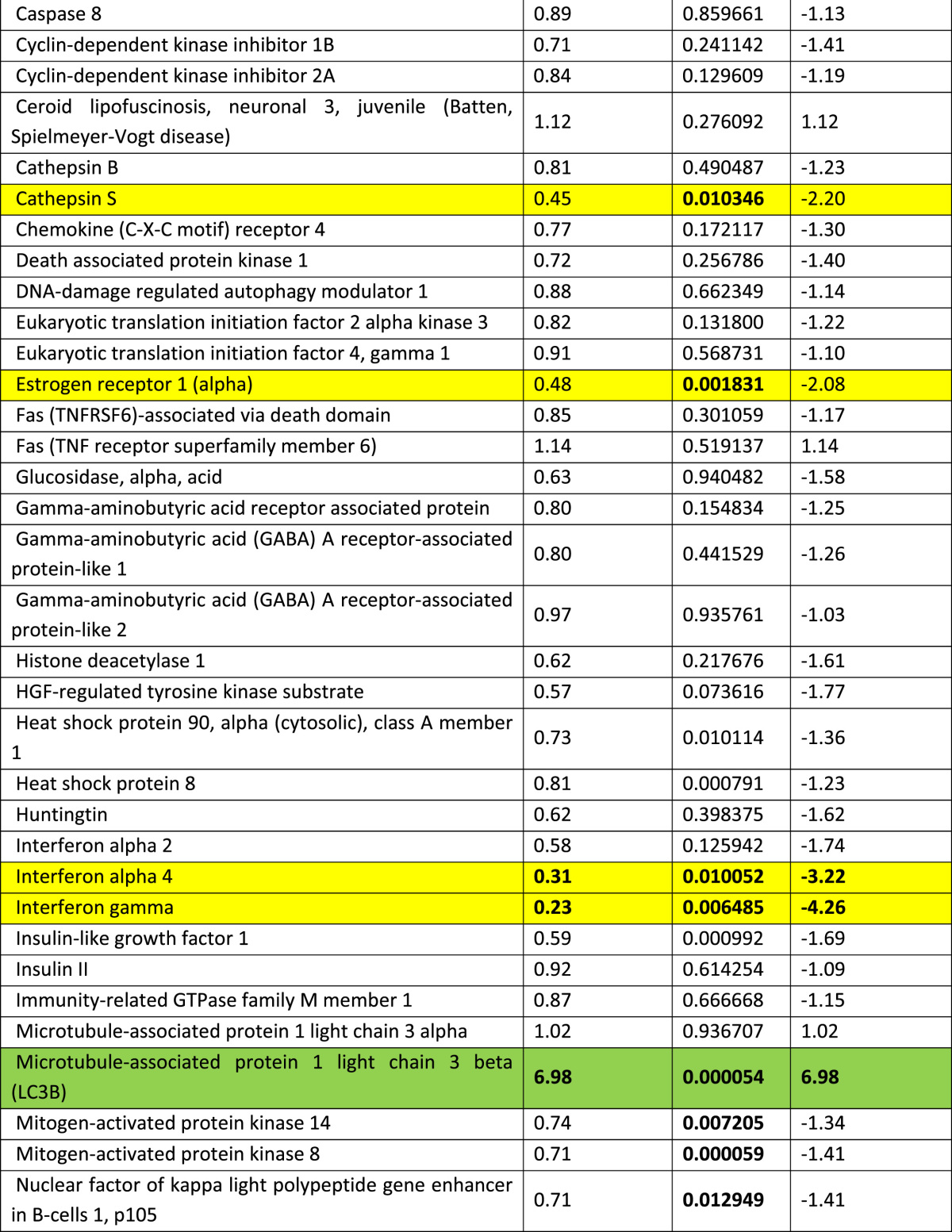
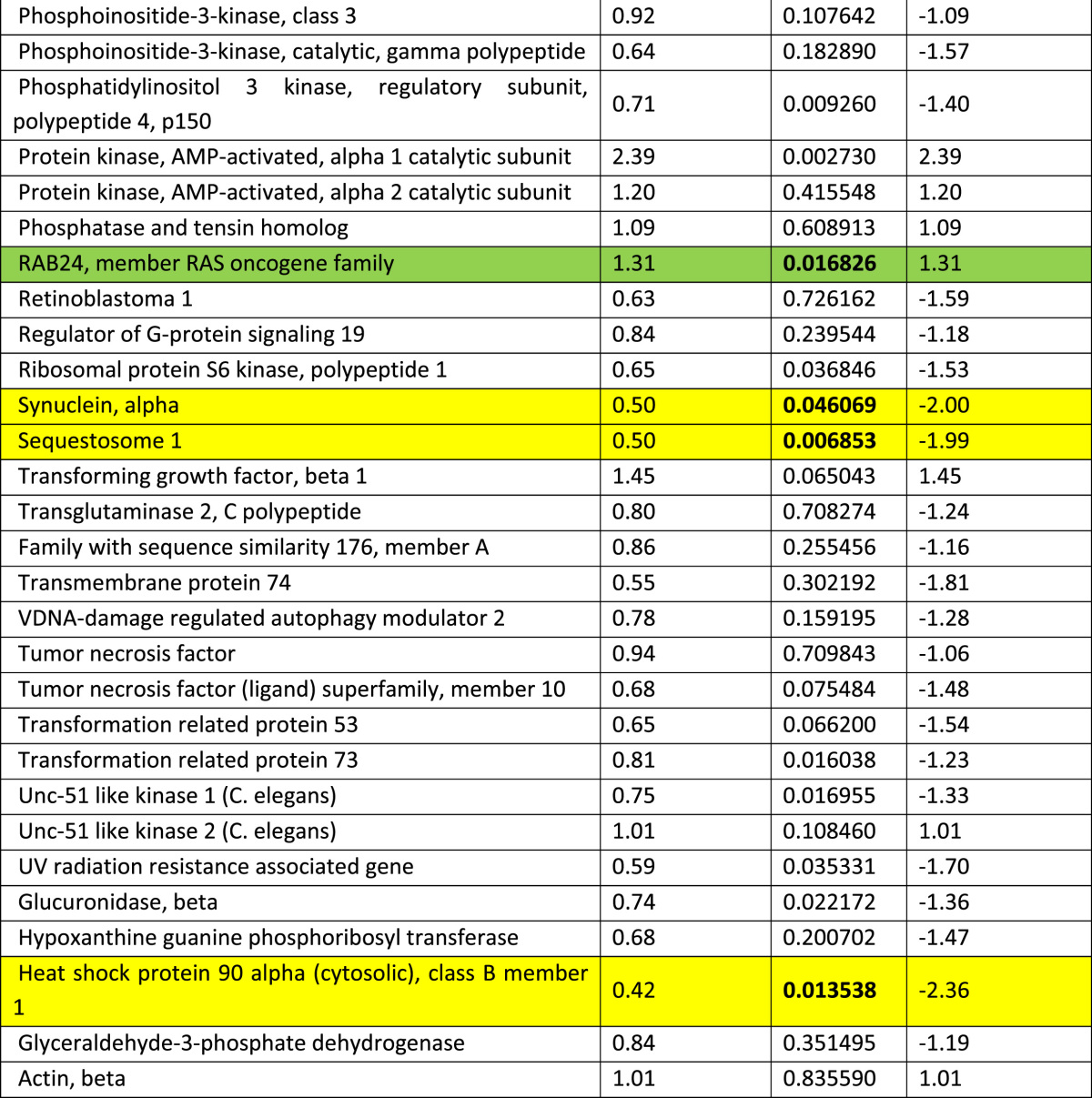

Highlighted in **green** are the significantly modulated autophagy related genes and highlighted in **yellow** are the significantly modulated cell survival related genes. Genes which are statistically significant (irrespective of the fold changes) are highlighted in red.
